# Randomized controlled trial: neostigmine for intra-abdominal hypertension in acute pancreatitis

**DOI:** 10.1186/s13054-022-03922-4

**Published:** 2022-03-03

**Authors:** Wenhua He, Peng Chen, Yupeng Lei, Liang Xia, Pi Liu, Yong Zhu, Hao Zeng, Yao Wu, Huajing Ke, Xin Huang, Wenhao Cai, Xin Sun, Wei Huang, Robert Sutton, Yin Zhu, Nonghua Lu

**Affiliations:** 1 Pancreatic Intensive Care Unit, Department of Gastroenterology, First Affiliated Hospital of Nanchang University, Nanchang, China; 2 Liverpool Pancreatitis Research Group, Institute of Systems, Molecular and Integrative Biology, University of Liverpool and Liverpool University Hospitals NHS Foundation Trust, Liverpool, Merseyside, UK; 3 Departments of Integrated Traditional Chinese and Western Medicine & Clinical Research Management, Sichuan Provincial Pancreatitis Center & West China-Liverpool Biomedical Research Center, West China Hospital, Sichuan University, Chengdu, China; 4 Chinese Evidence-Based Medicine Center and CREAT Group, West China Hospital, Sichuan University, Chengdu, China

**Keywords:** Neostigmine, Acute pancreatitis, Intra-abdominal hypertension, Acute compartment syndrome

## Abstract

**Background:**

Intra-abdominal hypertension (IAH) in acute pancreatitis (AP) is associated with deterioration in organ function. This trial aimed to assess the efficacy of neostigmine for IAH in patients with AP.

**Methods:**

In this single-center, randomized trial, consenting patients with IAH within 2 weeks of AP onset received conventional treatment for 24 h. Patients with sustained intra-abdominal pressure (IAP) ≥ 12 mmHg were randomized to receive intramuscular neostigmine (1 mg every 12 h increased to every 8 h or every 6 h, depending on response) or continue conventional treatment for 7 days. The primary outcome was the percent change of IAP at 24 h after randomization.

**Results:**

A total of 80 patients were recruited to neostigmine (*n* = 40) or conventional treatment (*n* = 40). There was no significant difference in baseline parameters. The rate of decrease in IAP was significantly faster in the neostigmine group compared to the conventional group by 24 h (median with 25th–75th percentile: −18.7% [− 28.4 to − 4.7%] vs. − 5.4% [− 18.0% to 0], *P* = 0.017). This effect was more pronounced in patients with baseline IAP ≥ 15 mmHg (*P* = 0.018). Per-protocol analysis confirmed these results (*P* = 0.03). Stool volume was consistently higher in the neostigmine group during the 7-day observational period (all *P* < 0.05). Other secondary outcomes were not significantly different between neostigmine and conventional treatment groups.

**Conclusion:**

Neostigmine reduced IAP and promoted defecation in patients with AP and IAH. These results warrant a larger, placebo-controlled, double-blind phase III trial.

*Trial registration* Clinical Trial No: NCT02543658 (registered August /27, 2015).

**Supplementary Information:**

The online version contains supplementary material available at 10.1186/s13054-022-03922-4.

## Introduction

Acute pancreatitis (AP) is a common disease of the digestive system [1]. Severe acute pancreatitis (SAP) with persistent organ failure is associated with an increased risk of death [2–5]. Intra-abdominal hypertension (IAH) is defined as a persistent increase in intra-abdominal pressure (IAP) ≥ 12 mmHg, according to the World Society for Abdominal Compartment Syndrome (WSACS) [6]. IAH is considered to be an early risk factor in the development of SAP [7]. In a prospective study, IAH was diagnosed in 17% of patients with AP, resulting in a mortality rate of 37% [8].

The inflammatory state of AP sparks a cascade of pancreatic and visceral edema, acute peripancreatic fluid collections, ascites, gut injury with paralytic ileus and gastric dilatation, leading to elevated IAP [9]. When IAP rises above 20 mmHg, abdominal compartment syndrome (ACS) and organ failure ensue [6]. AP complicated by ACS is associated with a mortality rate of 49% [9]. Although surgical decompression can promptly improve ACS, it causes substantial morbidity [9]. Thus, non-operative strategies for reducing IAH in AP patients are preferred, including nasogastric decompression, promotility agents and percutaneous catheter drainage (PCD), etc. [6, 7]. Neostigmine is an anti-cholinesterase drug that can enhance intestinal peristalsis, promoting the passage of flatus and defecation. Treatment with neostigmine effectively induces colonic decompression among those patients with colonic pseudo-obstruction [10–14]. The WSACS has suggested neostigmine be used for the treatment of established colonic ileus associated with IAH that does not respond to other simple measures [6]. No robust evidence exists, however, on the effects of pharmacological promotility therapy for IAP or outcomes among those with IAH/ACS [6]. Whether neostigmine can effectively reduce IAP and is beneficial in AP is unclear. In addition, confirmation of colonic ileus requires an abdominal X-ray typically in a standing position or computed tomography (CT) of the abdomen, likely to be unsuitable for patients with organ failure or hemodynamical instability during the early phase of AP. Paralytic ileus is a common risk factor for IAH in patients with AP [7]. If conventional treatment fails to correct IAH, there may be persistent paralytic ileus contributing to IAH, in which case neostigmine treatment may be beneficial.

This trial aimed to evaluate the efficacy of neostigmine in reducing IAP in patients with AP and persistent IAH following 24 h of conventional treatment, whether or not colonic pseudo-obstruction was established by X-ray or computerized tomography.

## Methods

### Study design and participants

This single-center, two-armed, parallel-group, superiority, randomized controlled phase II clinical trial was conducted between September 2015 and August 2017 in the Pancreatic Intensive Care Unit (ICU) of the Department of Gastroenterology at the First Affiliated Hospital of Nanchang University. This trial was registered (ClinicalTrials.gov, No. NCT02543658) and conducted adhering to a protocol that was approved by the Medical Ethics Research Committee of our hospital (No. 2015-011).

Patients aged between 18 and 70 years old who were within two weeks of AP onset and diagnosed with IAH during their Pancreatic ICU stay were assessed for eligibility. IAP was measured indirectly, using intravesicular pressure measured through a bladder catheter [6]. Briefly, the patient was in the supine position, 25 ml sterile normal saline was injected through the bladder before measurement, and with the transducer zeroed at the level where the midaxillary line crosses the iliac crest. For mechanically ventilated patients, IAP was measured under sedation. For patients with spontaneous breathing, IAP was measured at end expiration and ensuring that abdominal muscle contractions are absent.

When IAP remained ≥ 12 mmHg after conventional treatment (including sedation and analgesia, nasogastric decompression, glycerin enema for defecation, negative fluid balance and PCD for ascites) for 24 h, participants were considered to be enrolled in the trial when they met no exclusion criteria. Exclusion criteria included: (1) history of laparotomy; (2) intra-abdominal bleeding; (3) contraindications to neostigmine: angina pectoris, myocardial infarction, ventricular tachycardia, bradycardia, acute circulatory failure, epilepsy, bronchial asthma, mechanical intestinal obstruction, hyperthyroidism, serious arrhythmia, intestinal fistula or allergy to neostigmine; (4) urinary tract infection, or previous bladder surgery; (5) pregnancy or lactation. All patients or their legal representatives provided written informed consent before randomization.

### Randomization and concealment

Patients were enrolled in this trial by gastroenterologists who evaluated the study participants in the Pancreatic ICU. A statistician generated a randomization list with a computer program for use in sealed, opaque envelopes. The allocation sequence was concealed from the researchers. Once the patient was included in the study, the sealed envelope was opened by one of the study investigators to determine the treatment allocation. Participants were allocated to the neostigmine group or conventional group in a 1:1 ratio. As the dosing schedule of neostigmine depended on the response in an unpredictable manner, the clinicians, outcome assessors, and patients were not blinded from assignment to intervention.

### Intervention and follow-up

In the neostigmine group, patients received an initial dose of neostigmine (given intramuscularly within minutes of randomization) of 1 mg every 12 h. If there was no defecation after 12 h, the dose was increased to 1 mg every 8 h; if there was no defecation after 24 h, the dose was increased to 1 mg every 6 h. Neostigmine was stopped if the IAP dropped below 12 mmHg; otherwise, it was administered continuously for 7 days. Both the neostigmine and conventional groups received concomitant treatments as follows: (1) gastrointestinal decompression with a nasogastric and/or rectal tube; (2) paraffin oil and liquid soaked preparation of rheum officinale (rhubarb) and glauber salt by a nasogastric or nasojejunal tube. These traditional Chinese medicine components are widely used in China to alleviate gut dysmotility and have been shown to mitigate the severity of AP in patients [15, 16]; (3) glycerin enema to promote defecation; (4) PCD for ascites; (5) intravenous albumin, diuretics and when indicated renal replacement therapy for fluid overload; (6) sedation and analgesia to avoid agitation and patient-ventilator asynchrony.

Patients with IAP < 15 mmHg received enteral nutrition (EN) through a nasojejunal tube. The rate was initiated at 20 ml/h and increased gradually by 15 ml every 8 h to the goal rate (25–35 kcal/kg/d), depending on patient tolerance [17, 18]. EN was stopped temporarily when the IAP ≥ 15 mmHg and parenteral nutrition was initiated. When any patient’s IAP rose above 25 mmHg, or there were progressive organ dysfunction and fulminant ACS [19], a multidisciplinary seminar was held, including gastroenterologists, surgeons, interventional physicians and intensive care physicians, to decide whether to perform a surgical decompression. All patients were followed-up at 1, 3 and 6 months after discharge through the outpatient interview or telephone connection. Patient demographics, hospitalization and follow-up data were recorded on standardized case record forms by an investigator or coordinator who was unaware of study-group assignments.

### Outcomes

Definitions of the primary and secondary endpoints are displayed in Table 1. During the internal review process, we found neostigmine has the most significant effect in reducing IAP within 24 h. Therefore, instead of the “percent change of IAP from randomization to 7 days” that registered in ClinicalTrials.gov., we modified the primary endpoint as “percent change of IAP at 24 h after randomization”. We measured IAP at 3 h after randomization, then every 6 h for the following 3 days (72 h). After this period, IAP was measured as clinically indicated; patients who remained in Pancreatic ICU with IAP ≥ 12 mmHg had IAP measured every 6 h, while those transferred to general wards with normal IAP had IAP measured once every other day until 7 days after randomization. The following secondary endpoints were analyzed: (1) stool volumes at 24 h and 7 days after randomization; (2) timing of the start of EN; (3) deterioration of IAH; (4) new-onset ACS; (5) new-onset organ failure; (6) mortality for index hospital stay and within 6 months of follow-up; (7) other complications, adverse events and costs. Known local and systemic complications of AP, including new-onset organ failure occurring after neostigmine treatment or in the absence of neostigmine treatment, were not recorded as adverse events.

**Table 1:** Definition of the primary and secondary endpoints

Endpoint	Definition
IAH	A sustained or repeated pathological elevation in IAP ≥ 12 mmHg
IAH grade	Grade I, IAP 12–15 mmHg
	Grade II, IAP 16–20 mmHg
	Grade III, IAP 21–25 mmHg
	Grade IV, IAP > 25 mmHg
ACS	A sustained IAP > 20 mmHg (with or without an APP < 60 mmHg) that is associated with new organ dysfunction/failure
Increase in stool volume	Increase in 24 h stool volume on a designated day (day 1, day 2, day 3, day 5, and day 7) after randomization above the baseline 24 h stool volume before randomization
New-onset ACS	ACS occurring after randomization (not present at any time before it), assessed for up to 4 weeks
Deterioration of IAH	IAP that rebounds ≥ 5 mmHg or increases to ≥ 20 mmHg within 7 days after randomization
New-onset organ failure	Organ failure occurring after randomization (not present at any time before randomization)
Multiple-organ failure	Failure of two or more organs
Respiratory failure	PaO_2_/FiO_2_ ≤ 300, or requirement for mechanical ventilation
Circulatory failure	Circulatory systolic blood pressure < 90 mmHg, despite adequate fluid resuscitation, or requirement for inotropic catecholamine support
Renal failure	Creatinine level > 177 μmol/L after rehydration or new need for haemofiltration or hemodialysis
Timing of EN	Time from randomization to the initiation of tolerated EN
Intra-abdominal bleeding	Intra-abdominal bleeding that requires surgical, radiologic, or endoscopic intervention
Enterocutaneous or enteric fistula	Secretion of fecal material from a percutaneous drain or inflow into a necrotic cavity, either from small or large bowel, confirmed by endoscopy, imaging, or during surgery
Adverse event	The following events occurred during the use of neostigmine: drug eruption, ataxia, convulsions, coma, slurred speech, anxiety, fear, cardiac arrest, or other untoward events not characteristic of or expected from AP; diarrhea was excluded as this was part of the therapeutic effect to reduce IAP

*ACS* abdominal compartment syndrome, *APP* intraperitoneal perfusion pressure, *EN* enteral nutrition, *IAH* intra-abdominal hypertension, *IAP* intra-abdominal pressure

### Statistical analysis

The sample size was calculated from our observational data in which IAP decreased by 30% after treatment with neostigmine for 24 h (10 patients), compared to 5% after conventional treatment for 24 h (10 patients) (unpublished data), predicting an absolute reduction in IAP of 25%. Allowing for the possibility that neostigmine treatment might be better than conventional treatment and 10% loss to follow-up, we set the power at 80% and alpha at 5%, requiring a sample size of 40 patients in each of the two groups, i.e., a total of 80 cases. Primary and secondary endpoints were compared between treatment groups, and both intention-to-treat and per-protocol analyses were performed. Furthermore, patients with baseline IAP above 15 mmHg were selected for intention-to-treat subgroup analysis. Student’s *t*-test was performed for continuous variables with normal distribution, and the Kruskal–Wallis *H* test in the absence of normal distribution. IAP at each time point was analyzed as post values in the intervention group vs post values in the control group by ANCOVA. The *X*^*2*^ test or Fisher exact test was performed for categorical variables, and relative risk (RR) was calculated for dichotomous variables. Two-tailed *P* < 0.05 was considered statistically significant. The analyses were performed using the SPSS25.0 statistical software (IBM Corp, Armonk, NY).

## Results

### Participant characteristics

**Fig. 1:**
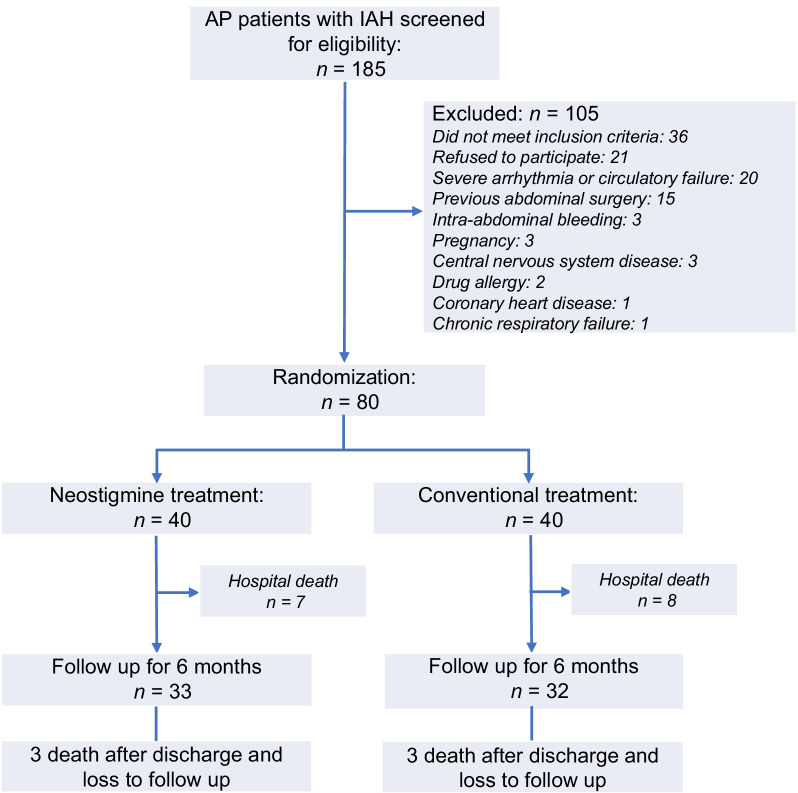
Study flowchart. AP, acute pancreatitis; IAH, intra-abdominal hypertension

From 1 September 2015 to 15 August 2017, 552 AP patients admitted to Pancreatic ICU in our hospital were screened, of whom 185 patients with IAH were assessed for eligibility and 80 patients were included and randomized to neostigmine (*n* = 40) or conventional (*n* = 40) treatment (Fig. 1). The etiology of AP in 41 (51%) patients was hypertriglyceridemia, defined as admission serum triglyceride level > 1000 mg/dL (11.3 mmol/L) and/or lipemic serum excluding other causes [20, 21], followed by 26 (32.5%) biliary and 8 (10%) alcohol excess. Before randomization, 38 (47.5%) patients had respiratory failure and 48 (60%) had IAP > 15 mmHg (15 (18.8%) with ACS). Four patients in the neostigmine group and 1 patient in the conventional group used opioids. In the neostigmine group, 3 patients had colonic ileus, 1 of whom used opioids.

Baseline characteristics were equally distributed between the two treatment groups (Table 2). There was no significant difference in baseline IAP between neostigmine and conventional groups (16.3 ± 2.7 vs. 15.9 ± 2.4, *P* = 0.63). In the neostigmine group, there were 33 (82.5%), 9 (22.5%) and 23 (57.5%) patients who received neostigmine every 12 h, for 3 days or for 7 days, respectively (Additional file 1: Table S1). In the conventional group, 4 patients were eventually given neostigmine because of a continuous increase in IAP. Therefore, separate per-protocol analyses were also conducted after excluding the aforementioned 4 patients. The baseline parameters remained comparable between the two groups in the per-protocol analysis (Additional file 1: Table S2).

**Table 2:** Intention-to-treat analysis of baseline characteristics

Characteristic	Neostigmine (*n* = 40)	Conventional (*n* = 40)	*P* value
Age (year)	46 ± 13	49 ± 14	0.85
Sex (m/f)	27/13	34/6	0.11
Etiology			
Biliary	12 (30.0%)	14 (35.0%)	0.95
Hypertriglyceridemia^a^	21 (52.5%)	20 (50.0%)	
Alcohol excess	4 (10.0%)	4 (10.0%)	
Idiopathic	3 (7.5%)	2 (5.0%)	
AP onset to hospital admission (d)	3 (1–4)	2 (1–3)	0.06
AP onset to randomization (d)	5 (3–7)	5 (4–6)	0.55
Comorbidity			
Diabetes mellitus	3 (7.5%)	6 (15.0%)	0.48
Hypertension	2 (5.0%)	7 (17.5%)	0.15
Coronary heart disease	1 (2.5%)	0	1.00
Chronic renal insufficiency	0	1 (2.5%)	1.00
Admission clinical severity			
SIRS	2 (2–3)	2 (2–3)	0.70
APACHE II	9 (7–9)	9 (7–12)	0.79
C-reactive protein (mg/L)	228.6 ± 144.1	295.8 ± 125.8	0.70
White cell count (× 10^9^/L)	14.7 ± 5.9	14.2 ± 5.6	0.45
Procalcitonin (ng/mL)	1.7 (0.6–13.7)	2.8 (1.3–6.7)	0.40
Serum lactate	2.0 ± 1.3	1.7 ± 0.9	0.16
Organ failure^b^	32 (80.0%)	27 (67.5%)	0.31
Single organ failure			
Respiratory	21 (52.5%)	17 (42.5%)	0.50
Renal	3 (7.5%)	1 (2.5%)	0.61
Multiple organ failure	8 (20.9%)	9 (22.5%)	1.00
CTSI within 1 week of AP onset^c^	5 (3–7)	5 (3–7)	0.99
ANC	28 (73.7%)	26 (76.4%)	0.63
APFC	10 (26.3%)	8 (23.5%)	0.59
IAH level before randomization, mmHg	16.3 ± 2.7	15.9 ± 2.4	0.63
Grade I	15 (37.5%)	17 (42.5%)	
Grade II	22 (55.0%)	21 (52.5%)	
Grade III	3 (7.5%)	2 (5.0%)	
Grade IV	0	0	
ACS	9 (22.5%)	6 (15.0%)	0.56
Use of opioids	4 (10.0%)	1 (2.5%)	0.36
Colonic ileus^c,d^	3 (7.9%)	0	0.11
24 h of defecation (mL)	450 (10–1050)	800 (520–990)	0.14
PCD of ascites	10 (25.0%)	6 (15.0%)	0.40
Admitted to the ICU at randomization	40 (100%)	40 (100%)	1.00

*ACS* abdominal compartment syndrome, *AP* acute pancreatitis, *APACHE II* acute physiology and chronic health evaluation II, *APFC* acute peripancreatic fluid collection, *ANC* acute necrotic collection, *CTSI* computed tomography severity index, *IAH* intra-abdominal hypertension, *ICU* Intensive Care Unit, *PCD* percutaneous catheter drainage, *RAC* Revised Atlanta Classification, *SAP* severe acute pancreatitis, *SIRS* systemic inflammatory response syndrome

^a^Defined as admission serum triglyceride level > 1000 mg/dL and/or lipemic serum after ruling out biliary and alcohol excess etiologies

^b^Patients with circulatory failure were excluded because neostigmine may affect the circulation

^c^There were 38 and 34 cases in the neostigmine group and conventional group, respectively, underwent CT within the first week after AP onset

^d^Opioids were used in 2 of the 3 patients with colonic ileus

### Intra-abdominal pressure after randomization

The IAP decreased after randomization in both groups, but dropped significantly faster as assessed at multiple time points in the neostigmine group than the conventional group (Table 3). IAP decreased from 16.3 ± 2.7 to 13.8 ± 3.5 mmHg after 9 h of neostigmine administration. IAP levels were significantly lower in neostigmine group than conventional treatment group at 9 h (13.8 ± 3.5 vs. 15.0 ± 3.1, *P* = 0.038), 15 h (13.3 ± 3.4 vs. 14.7 ± 3.1, *P* = 0.015), and 168 h (12.2 ± 2.7 vs. 13.6 ± 3.5, *P* = 0.045) after randomization.

**Table 3:** Intra-abdominal pressure from randomization to 7 days

Time (h)	Intention-to-treat analysis			Subgroup analysis (IAP > 15 mmHg at baseline)			Per-protocol analysis		
	Neostigmine (*n* = 40)	Conventional (*n* = 40)	*P* value^†^	Neostigmine (*n* = 25)	Conventional (*n* = 23)	*P* value^†^	Neostigmine (*n* = 40)	Conventional (*n* = 36)	*P* value^†^
	IAP	IAP		IAP	IAP		IAP	IAP	
0	16.3 ± 2.7	15.9 ± 2.4		17.9 ± 2.0	17.6 ± 1.7		16.3 ± 2.7	15.9 ± 2.5	
3	14.6 ± 3.0	15.0 ± 3.1	0.205	15.0 ± 3.0	16.7 ± 2.6	0.010	14.6 ± 3.0	14.9 ± 2.9	0.322
9	13.8 ± 3.5	15.0 ± 3.1	0.038	14.2 ± 3.3	16.0 ± 3.4	0.018	13.8 ± 3.5	14.7 ± 2.8	0.079
15	13.3 ± 3.4	14.7 ± 3.1	0.015	13.2 ± 3.7	15.8 ± 3.0	0.001	13.3 ± 3.4	14.7 ± 3.1	0.015
24	13.7 ± 3.6	14.7 ± 3.2	0.083	13.7 ± 3.6	15.2 ± 3.2	0.020	13.7 ± 3.6	14.5 ± 3.1	0.152
30	13.7 ± 3.5	14.3 ± 3.1	0.323	13.4 ± 3.2	15.0 ± 2.9	0.038	13.7 ± 3.5	14.0 ± 3.1	0.533
36	14.3 ± 3.6	13.8 ± 3.0	0.619	14.2 ± 3.5	14.1 ± 3.1	0.849	14.3 ± 3.6	13.7 ± 3.0	0.521
42	13.8 ± 3.1	14.2 ± 2.6	0.454	13.7 ± 3.4	14.5 ± 2.4	0.183	13.8 ± 3.1	14.2 ± 2.5	0.465
48	14.1 ± 3.2	13.7 ± 2.9	0.767	14.8 ± 3.0	14.4 ± 2.2	0.895	14.1 ± 3.2	13.4 ± 2.9	0.489
54	13.6 ± 3.4	13.4 ± 2.8	0.961	14.1 ± 3.4	14.0 ± 2.1	0.845	13.6 ± 3.4	13.2 ± 2.6	0.790
60	13.0 ± 3.1	13.6 ± 2.6	0.280	13.0 ± 3.2	14.1 ± 2.1	0.097	13.0 ± 3.1	13.3 ± 2.4	0.522
66	13.6 ± 3.1	13.3 ± 3.9	0.853	14.0 ± 2.8	13.7 ± 3.4	0.904	13.6 ± 3.1	12.9 ± 3.7	0.468
72	13.2 ± 2.9	13.8 ± 2.9	0.237	13.8 ± 3.1	14.2 ± 2.4	0.490	13.2 ± 2.9	13.4 ± 2.6	0.589
120	13.2 ± 3.2	13.8 ± 2.9	0.213	13.6 ± 3.2	14.4 ± 2.8	0.261	13.2 ± 3.2	13.2 ± 2.2	0.731
168	12.2 ± 2.7	13.6 ± 3.5	0.045	11.9 ± 2.8	14.2 ± 3.6	0.013	12.2 ± 2.7	13.0 ± 2.7	0.199

IAP, intra-abdominal pressure

^†^IAP at each time point were analyzed as post values in the intervention group versus post values in the control group by ANCOVA

Subgroup analysis restricting patient inclusion to baseline IAP > 15 mmHg showed that the mean IAP levels in the neostigmine group were significantly lower than conventional group at 3 h, 9 h, 24 h, 30 h, and 168 h after randomization (all *P* ≤ 0.038) (Table 3). In per-protocol analysis, IAP at 15 h remained markedly lower in neostigmine group (*P* = 0.015) (Table 3).

### Primary outcome

Intension-to-treat analysis (Table 4) showed that the rate of decrease in IAP was significantly faster in the neostigmine group compared to the conventional group by 24 h (median with 25th–75th percentile: − 18.7% [− 28.4 to − 4.7%) vs − 5.4% [− 18.0% to 0], *P* = 0.017). Intension-to-treat subgroup analysis of AP patients with baseline IAP > 15 mmHg and per-protocol analysis also showed that IAP decreased significantly faster in the neostigmine group than in the conventional group (Additional file 1: Tables S3 and S4).

**Table 4:** Intention-to-treat analysis of primary endpoint and secondary endpoints

Endpoint	Neostigmine (*n* = 40)	Conventional (*n* = 40)	RR (95% CI)	*P* value
*Primary endpoint*				
Percent change of IAP at 24 h, %	− 18.7 ([− 28.4]-[− 4.7])	− 5.4 ([− 18.0]− 0)		0.017
*Secondary endpoint*				
Increase in stool volume at 24 h after randomization (mL)	870 (250–2070)	60 ([− 30]− 770)		0.00
Increase in stool volume at 7 d after randomization (mL)	1025 (450–1520)	370 (150–1200)		0.02
Serum lactate	1.7 ± 0.8	1.6 ± 0.7		0.97
Timing of EN^a^	2 (0–3)	2 (0–3)		1.00
Deterioration of IAH^b^	4 (10.0%)	8 (20.0%)	0.50 (0.16–1.53)	0.35
New-onset ACS	2 (5.0%)	4 (10.0%)	0.50 (0.10–2.58)	0.68
New-onset organ failure	12 (30.0%)	16 (40.0%)	0.75 (0.41–1.38)	0.48
Single organ failure				
Respiratory	2 (5.0%)	6 (15.0%)	0.33 (0.07–1.55)	0.26
Circulatory	3 (7.5%)	3 (7.5%)	1.00 (0.21–4.66)	1.00
Renal	0	3 (7.5%)	–	0.24
Multiple organ failure	7 (15.0%)	4 (10.0%)	1.75 (0.56–5.52)	0.52
Invasive interventions^c^				
Percutaneous catheter drainage	8 (20.0%)	5 (12.5%)	1.60 (0.58–4.48)	0.55
Endoscopic transmural drainage	3 (7.5%)	4 (10.0%)	0.75 (0.18–3.04)	1.00
Endoscopic necrosectomy^d^	1 (2.5%)	2 (5.0%)	0.50 (0.05–5.30)	1.00
Surgical laparotomy	3 (7.5%)	4 (10.0%)	0.75 (0.18–3.14)	1.00
Intra-abdominal bleeding (requiring intervention)	2 (5.0%)	4 (10.0%)	0.50 (0.10–2.58)	0.68
Enterocutaneous fistula (requiring intervention)	2 (5.0%)	0	–	0.49
Septicemia	11 (27.5%)	11 (27.5%)	1.00 (0.49–2.04)	1.00
Vascular complications^e^	4 (11.8%)	5 (13.5%)	0.87 (0.26–2.98)	0.56
Portal vein thrombosis	0	3 (7.5%)		0.24
Splenic vein thrombosis / splenic infarction	3 (7.5%)	1 (2.5%)		0.53
Portal vein and splenic vein thrombosis	0	1 (2.5%)		0.49
Superior mesenteric vein and splenic vein thrombosis	1 (2.5%)	0		0.49
RAC disease severity				
MSAP	3 (7.5%)	5 (12.5%)	0.60 (0.15–2.34)	0.71
SAP	37 (92.5%)	35 (87.5%)	1.05 (0.91–1.22)	0.71
Death in index hospital stay	7 (17.5%)	8 (20%)	0.88 (0.35–2.18)	0.77
Length of ICU stay (d)	14 ± 9	15 ± 14		0.94
Length of hospital stay (d)	23 ± 13	22 ± 16		0.48
Medical expenses (1000 RMB)	125.9 ± 83.6	134.3 ± 128.8		0.80
*Follow-up (6 M)*	*N* = 33	*N* = 32		
Pancreatic pseudocyst	2 (6.1%)	1 (3.1%)	1.94 (0.18–20.35)	1.00
Needing elective intervention	0	1 (3.1%)	–	0.49
Walled-off necrosis	14 (42.4%)	11 (34.4%)	1.23 (0.66–2.30)	0.61
Needing elective intervention	3 (9.1%)	1 (3.1%)	2.91 (0.32–26.52)	0.61
Portal thrombosis	1 (3.1%)	1 (3.1%)	0.97 (0.06–14.85)	1.00
Pancreatogenic portal hypertension	1 (3.1%)	2 (6.1%)	0.48 (0.04–5.62)	1.00
New onset diabetes	9 (27.3%)	5 (15.6%)	2.03 (0.60–6.88)	0.37
Impaired glucose tolerance	3 (9.1%)	2 (6.3%)	1.55 (0.23–19.63)	1.00
External secretion dysfunction	7 (24.1%)	4 (13. 3%)	2.06 (0.53–8.00)	0.33
Recurrent AP	4 (12.2%)	1 (3.1%)	4.28 (0.45–40.53)	0.36
Death after discharge	3 (9.1%)	3 (9.4%)	0.97 (0.18–5.19)	1.00

*ACS* abdominal compartment syndrome, *AP* acute pancreatitis, *CI* confidence interval, *EN* enteral nutrition, *IAH* intra-abdominal hypertension, *ICU* Intensive Care Unit, *MSAP* moderately severe acute pancreatitis, *RR* relative risk, *SAP* severe acute pancreatitis

^a^Time from randomization to initiation of EN

^b^IAP that rebounded ≥ 5 mmHg or increased ≥ 20 mmHg in 1–7 days after randomization

^c^All interventions after randomization were counted

^d^In the neostigmine group, 1 case underwent endoscopic debridement; in the conventional group, 1 case underwent percutaneous retroperitoneal endoscopic debridement and 1 case underwent endoscopic debridement

^e^30 cases in the neostigmine group, and 32 cases in conventional group received CT enhancement and vascular imaging

### Secondary outcomes

**Fig. 2:**
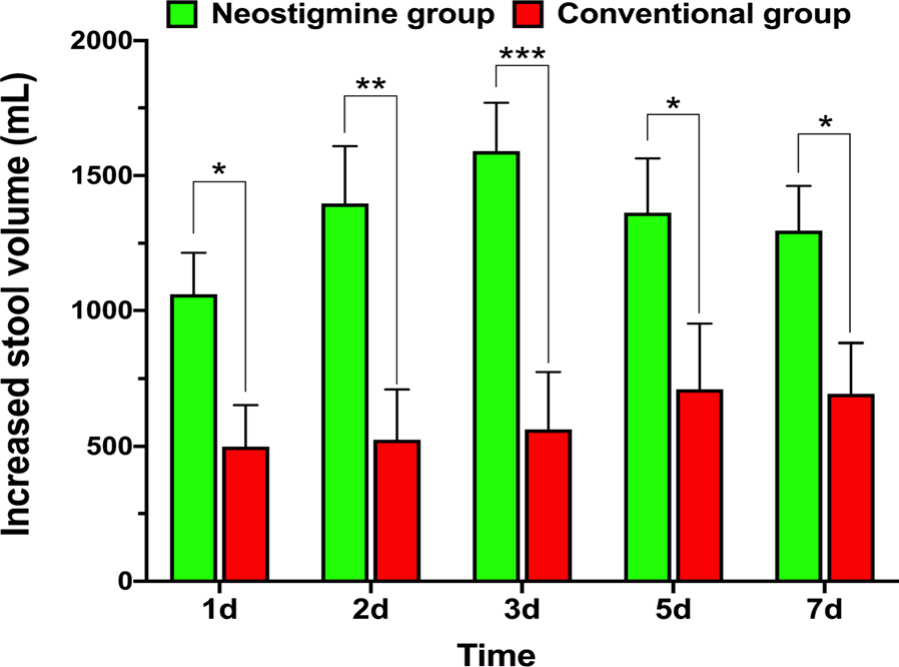
Intention-to-treat plots of increased stool volumes (mL/24 h minus baseline 24 h stool volume before randomization). *P < 0.05, **P < 0.01, ***P < 0.001)

Stool volumes (above baseline) were consistently higher in the neostigmine group compared to the conventional group from day 1 (median with 25th–75th percentile: 870 ml [250–2070] vs. 60 ml [30–770], *P* = 0.00) to day 7 [1025 ml [450–1520] vs. 370 ml [150–1200], *P* = 0.02; Fig. 2 and Table 4). The timing of EN (2 [0–3] vs. 2 [0–3] days), deterioration of IAH (4/40 vs. 8/40), new-onset ACS (2/40 vs 4/40), new-onset organ failure (12/40 vs. 16/40), mortality (7/40 vs. 8/40) and other outcome measures including follow-up for 6 months were not significantly different. These results remained unchanged by per-protocol analysis (Additional file 1: Table S4). In the subgroup analysis for baseline IAP > 15 mmHg, only stool volumes (above baseline) at day 1 were higher in the neostigmine group compared to the conventional group; all the remaining results were not statistically different between the two groups (Additional file 1: Table S3).

Among 12 patients with worsening IAH, 3 patients had intra-abdominal bleeding, 2 of whom underwent successful hemostasis and IAH subsequently decreased. Hemostasis could not be achieved in 1 patient who eventually died from uncontrollable progressive ACS. Five of the 12 patients with worsening IAH were fluid overloaded, so diuretics, albumin and/or renal replacement therapy were instituted: IAP reduced in 1, who survived; 2 developed ACS and multiple organ failure and died, while 2 had persistent respiratory failure, sepsis and died. The remaining 4 patients with worsening IAH had > 50% pancreatic necrosis and fluid accumulation in the retroperitoneum: 1 did not develop ACS or infection and survived, while 3 developed ACS, infected pancreatic necrosis, multiple organ failure and died.

### Adverse events

Neostigmine treatment was considered unlikely to be related to all 6 adverse events (Additional file 1: Tables S5 and S6). The new-onset circulatory failure occurred in 3 patients in the neostigmine group and 1 patient in the conventional group given neostigmine because of continuous increase in IAP. All 4 had respiratory and/or renal failure prior to randomization, and circulatory failure was attributed to progression of AP. One patient in the neostigmine group developed new-onset respiratory failure after rebound elevation in IAP leading to ACS. Overall, 12 patients in the neostigmine group compared to 16 patients in the conventional group developed new-onset organ failure after randomization (new-onset organ failure after cessation or in the absence of neostigmine administration was not recorded as an adverse event). One patient in the neostigmine group developed bradycardia while also receiving esmolol, a cardio-selective beta_1_ receptor blocker with rapid onset and short duration of action; the bradycardia ceased after withdrawal of esmolol during continuation of neostigmine.

## Discussion

This first randomized controlled trial of neostigmine as a promotility agent for AP patients with IAH/ACS showed neostigmine treatment was significantly more effective than conventional treatment in reducing IAP in AP patients with persistent IAH after 24 h of conventional treatment (including gastrointestinal decompression, glycerin enema, negative fluid balance, and ascites drainage). The decrease in IAP occurred within 3 h of neostigmine administration and was most pronounced within the first 15 h and more evident in patients with severe IAP (> 15 mmHg) at baseline. The reduction in IAP appears relatively modest in neostigmine group; the first reason is that the patients included in this study were difficult to reduce the IAP to normal after 24 h of other simple measures; secondly, for ethical reasons, patients in the conventional group also used Chinese traditional medicine—rheum officinale and glauber salt to promote gastrointestinal peristalsis after randomization. A small pilot study on SAP patients found that dachengqi decoction with rheum officinale and glauber salt as the main components can significantly reduce IAP on days 4–8 after admission [11]. We believe that the use of neostigmine in patients with IAH above grade II (> 15 mmHg) is more clinically significant, since the effect of neostigmine on reducing IAP is more profound in patients with IAP greater than 15 mmHg. Subclinical organ injury develops at levels of IAP previously deemed to be safe (IAP between 12 and 15 mmHg), but as IAP increases, organ dysfunction will become more pronounced, and there is a dose-dependent relationship between IAP and organ dysfunction [22].

As IAH/ACS is associated with a higher incidence of organ dysfunction [23], higher mortality and longer hospitalization in patients with SAP [24], reducing IAP as promptly as possible may improve outcomes in AP. We found no statistically significant difference, however, in the incidence of new organ failure or other complications between neostigmine and conventional treatment, although patients receiving neostigmine showed a trend toward less new-onset respiratory (2 of 40 vs. 6 of 40), renal (0 vs. 3) and overall organ (12 vs. 16) failure.

Neostigmine treatment resulted in significantly increased stool volumes compared to conventional treatment, confirming enhanced intestinal peristalsis, although this did not significantly hasten the initiation of EN. Early EN has been shown to enhance recovery in AP, likely by protecting the gut mucosal barrier and reducing bacterial translocation, infected pancreatic necrosis and other severe outcomes [25]. The optimal time to initiate EN in patients with AP is considered to be within 24–72 h of admission, allowing for early intolerance to oral feeding [26]. IAH/ACS impedes early EN and is commonly associated with food intolerance. A prospective pilot study found early EN to hinder the development of IAH and reduce the severity of SAP compared with delayed EN [17]. In our study, neostigmine treatment promoted gut motility and thus reduced IAP, which may increase tolerance to EN. The median time to initiation of EN in the neostigmine and conventional groups was, however, comparable at the second day after randomization (third day after admission to Pancreatic ICU), within the period recommended in guidelines [27]. European Society of Intensive Care Medicine (ESICM) clinical practice guidelines recommend EN should be administered with caution when IAP reaches ≥ 15 mmHg [27]. The high proportion (48, i.e., 60%) of our patients who had IAP ≥ 15 mmHg at entry into a trial may partially explain the lack of any significant effect of neostigmine on the timing of EN initiation.

Neostigmine blocks the active site of anticholinesterase, increasing the availability of acetylcholine to ligate nicotinic ion channel receptors and muscarinic G-protein receptors that elicit second messenger cascades [28]. Muscarinic receptors predominate in the enteric nervous system, which response to vagal parasympathetic preganglionic activation, as well as operating independently to drive motility, secretion, the cholinergic anti-inflammatory pathway, epithelial proliferation and repair [28, 29]. Vagal nerve stimulation has shown consistent anti-inflammatory effects in colitis models, whereas vagotomy increases inflammatory markers in these models [28] and greater severity of pancreatic injury in experimental AP [30]. The evidence from our trial indicates a direct beneficial effect of neostigmine on the gut function that may include reduction in gut injury, which might contribute to improved outcomes from AP. Despite the predominance of hypertriglyceridemia etiology in our trial (41 of 80 patients), which has a high incidence in China [31–34], and induces more SAP [20, 34, 35], the mechanisms of action of neostigmine are likely to apply equally to other etiologies of human AP, although this remains to be tested further.

Patients in both the neostigmine and conventional groups were given rheum officinale (rhubarb) and glauber salt to promote defecation. Rhubarb and glauber salt are principal constituents of dachengqi decoction and its derivatives, commonly used in China to treat AP [15, 16], but they are rarely used in other countries in the world. Modified dachengqi decoction significantly decreased IAP 4–8 days after admission and improved clinical outcomes in a randomized trial conducted in predicted SAP patients [36]. A recent meta-analysis [37] of 11 randomized trials has shown that the combination of rhubarb and early EN compared to early EN alone improves gut motility, enhances the efficacy of EN and reduces AP severity in predicted SAP patients. Despite the use of rhubarb and glauber salt in both our treatment groups; however, neostigmine had significant beneficial effects on IAP and gut function, indicative of an independent action that may be widely applicable to patients with AP and IAH ± ACS.

Neostigmine was not effective in reducing IAH in all AP patients. In the neostigmine group, IAP rebounded by ≥ 5 mmHg or rose to > 20 mmHg in 4 patients, 2 of whom developed new ACS. In addition to ileus, distention, inflammation and gut wall edema, extra-luminal factors may contribute to the development of IAH in AP. These factors include third space fluid losses, acute fluid collections, fluid overload, pancreatic/peri-pancreatic necrosis and/or intra-abdominal hemorrhage, each of which may contribute to the failure of treatment for IAH, notwithstanding additional water losses in stool from neostigmine treatment. A previous randomized controlled trial found controlled fluid resuscitation reduced the incidence of ACS in patients with SAP [38], although optimal protocols are yet to be worked out.

In this study, we did not observe any adverse event likely to be related to neostigmine treatment, but a potential safety signal remains, since neostigmine may increase cardiac output by lowering IAP and thus systemic vascular resistance, yet result in an increased cholinergic drive that may slow heart rate [13]. Symptomatic bradycardia has been observed in patients treated with neostigmine for acute colonic pseudo-obstruction, but the bradycardia that developed in our patient resolved on cessation of the β-adrenergic antagonist esmolol. Patients with underlying brady-arrhythmias or those receiving β-adrenergic antagonists are likely to be more susceptible to bradycardia during the treatment of neostigmine [10]. In addition to bradycardia, neostigmine may also cause bronchoconstriction and increase airway resistance [13], although this has not been observed in previous studies [10–13] and was not in our study. A further caution would be the late use of neostigmine 4–6 weeks after the onset of AP, because of a risk of acute or sub-acute intestinal obstruction in the presence of adhesions caused by fibrosis of necrotic tissue.

### Limitations

This trial was not blinded, allowing for flexibility to alter the frequency of neostigmine administration depending on changes in IAP, but introducing potential bias, e.g., timing the start of EN. As neostigmine treatment every 12 h was sufficient for the vast majority of (35 of 40) patients, this frequency or specific rules could be adopted in any future double-blind trial. Our trial had a relatively small sample size, designed as a phase II trial to test the efficacy of neostigmine on the reduction in IAP, but not on the complications of AP.

## Conclusion

This preliminary study found neostigmine to reduce IAP effectively in patients with AP and IAH who were not responding to conventional treatment, by enhancing intestinal peristalsis and promoting defecation, especially in patients with baseline IAP > 15 mmHg. These results warrant a larger, multi-center, phase III trial designed to assess the impact of neostigmine on the complications of AP from multiple etiologies.

## Supplementary Information

**Additional file 1: Table S1. **Schedule of neostigmine administration in the neostigmine group: (**a**) frequency and (**b**) duration, **Table S2.** Per-protocol analysis of baseline characteristics, **Table S3. **Per-protocol analysis of secondary endpoints, **Table S4.** Subgroup analysis of secondary endpoints (IAP ≥ 15 mmHg at randomization), **Table S5. **Characteristics of patients who developed adverse events, **Table S6. **Adverse events, causes and outcomes.

## Data Availability

The datasets used and/or analyzed during the current study are available from the corresponding author on reasonable request.

## Abbreviations

ACS: Abdominal compartment syndrome
AP: Acute pancreatitis
CT: Computed tomography
EN: Enteral nutrition
IAH: Intra-abdominal hypertension
IAP: Intra-abdominal pressure
PCD: Percutaneous catheter drainage
SAP: Severe acute pancreatitis
