# Supplementary material for: Randomized controlled trial: neostigmine for intra-abdominal hypertension in acute pancreatitis
Source: Crit Care. 2022 Mar 3;26:52. doi: 10.1186/s13054-022-03922-4 (PMC8892692; doi:10.1186/s13054-022-03922-4)
Supplement: Supplementary file 1 — Additional file 1: Table S1. Schedule of neostigmine administration in the neostigmine group: (a) frequency and (b) duration, Table S2. Per-protocol analysis of baseline characteristics, Table S3. Per-protocol analysis of secondary endpoints, Table S4. Subgroup analysis of secondary endpoints (IAP ≥ 15 mmHg at randomization), Table S5. Characteristics of patients who developed adverse events, Table S6. Adverse events, causes and outcomes. [file 13054_2022_3922_MOESM1_ESM.docx]

**Additional file 1: Table S1. Schedule of neostigmine administration in the neostigmine group: (a) frequency and (b) duration.**

**a**

| **Frequency** | **No. of patients** |
| --- | --- |
| Single dose | 2 (5.0%) |
| Every 12 h (q12h) | 33 (82.5%) |
| Every 8 h (q8h) | 3 (7.5%) |
| Every 6 h (q6h) | 2 (5.0%) |

**b**

| **Duration (d)** | **No. of patients** |
| --- | --- |
| 1 | 1 (2.5%) |
| 2 | 0 |
| 3 | 9 (22.5%) |
| 4 | 2 (5.0%) |
| 5 | 3 (7.5%) |
| 6 | 2 (5.0%) |
| 7 | 23 (57.5%) |

**Table S2. Per-protocol analysis of baseline characteristics**

| **Characteristic** | | |  | **Neostigmine**  **(n = 40)** | **Conventional**  **(n = 36)** | ***P***  **value** |
| --- | --- | --- | --- | --- | --- | --- |
| Age (yr) | | | | 46 ± 13 | 50 ± 15 | 0.28 |
| Sex (m/f) | | | | 27/13 | 30/6 | 0.18 |
| Etiology | | | |  |  | 0.87 |
|  | Biliary | |  | 12 (30.0%) | 14 (38.9%) |  |
|  | Hypertriglyceridemia^†^ | | | 21 (52.5%) | 17 (47.2%) |  |
|  | Alcohol excess | | | 4 (10.0%) | 3 (8.3%) |  |
|  | Idiopathic | | | 3 (7.5%) | 2 (5.6%) |  |
| AP onset to hospital admission (d) | | | | 3 (1-4) | 2 (1-3) | 0.10 |
| AP onset to randomization (d) | | | | 5 (3-7) | 5 (4-6) | 0.54 |
| Comorbidity | | | |  |  |  |
|  | Hypertension | | | 2 (5.0%) | 6 (16.72%) | 0.14 |
|  | Diabetes mellitus | |  | 3 (7.5%) | 5 (13.9%) | 0.4 |
|  | Coronary heart disease | | | 1 (2.5%) | 0 | 1.00 |
|  | Chronic renal insufficiency | | | 0 | 1 (2.8%) | 0.47 |
| Admission clinical severity score | | | |  |  |  |
| SIRS | | | | 2 (2-3) | 2 (2-3) | 0.44 |
| APACHE II | | | | 9 (7-13) | 9 (6-12) | 0.68 |
| Admission biochemical index | | | |  |  |  |
|  | C-reactive protein, mg/L | | | 228.6 ± 144.1 | 287.3 ± 127.9 | 0.10 |
|  | White cell count (×10^9^/L) | | | 14.7 ± 5.9 | 14.5 ± 5.7 | 0.90 |
|  | Procalcitonin (ng/mL) | | | 1.8 (0.6-13.7) | 2.3 (0.8-5.7) | 0.72 |
| Organ failure^‡^ | | | |  |  |  |
| Single organ failure | | | |  |  |  |
| Respiratory | | | | 21 (52.5%) | 16 (44.4%) | 0.50 |
| Renal | | | | 3 (7.5%) | 1 (2.8%) | 0.62 |
| Multiple organ failure | | | | 8 (20.0%) | 6 (16.7%) | 0.77 |
| CTSI within 1 week of onset^§^ | | | | 5 (3-7) | 5 (3-7) | 0.72 |
| ANC | | | | 28 (70.0%) | 22 (61.1%) | 0.47 |
| APFC | | | | 10 (25.0%) | 8 (22.2%) | 0.79 |
| IAH level at randomization, mmHg | | | | 16.3 (2.7) | 15.9 (2.5) | 0.59 |
|  | Grade I |  | | 15 (37.5%) | 16 (44.4%) |  |
|  | Grade II |  | | 22 (55.0%) | 18 (50.0%) |  |
|  | Grade III |  | | 3 (7.5%) | 2 (5.6%) |  |
|  | Grade IV |  | | 0 | 0 |  |
| ACS | | | | 9 (22.5%) | 4 (11.1%) | 0.23 |
| 24 h of defecation (mL) | | | | 450 (10-1050) | 800 (610-950) | 0.16 |
| PCD of ascites | | | | 10 (25.0%) | 5 (13.9%) | 0.26 |
| Admitted to the ICU at randomization | | | | 40 (100%) | 36 (100%) | 1.00 |

ACS, abdominal compartment syndrome; AP, acute pancreatitis; APACHE II, Acute Physiology and Chronic Health Evaluation II; APFC, acute peripancreatic fluid collection; ANC, acute necrotic collection; CTSI, computed tomography severity index; IAH, intra-abdominal hypertension; ICU, Intensive Care Unit; PCD, percutaneous catheter drainage; RAC, Revised Atlanta Classification; SAP, severe acute pancreatitis; SIRS, Systemic Inflammatory Response Syndrome.

^†^Defined as admission serum triglyceride level > 1000 mg/dL and/or lipemic serum after ruling out biliary and alcohol excess etiology.

^‡^Patients with circulatory failure were excluded because neostigmine may affect the circulation.

^§^There were 38 and 34 cases in the neostigmine group and conventional group, respectively, underwent CT within the first week after AP onset.

**Table S3. Subgroup analysis of of primary endpoint and secondary endpoints**

| **Endpoint** | **Neostigmine**  **(n = 25)** | **Conventional**  **(n = 23)** | **RR (95% CI)** | ***P***  **value** |
| --- | --- | --- | --- | --- |
| **Primary endpoint** |  |  |  |  |
| Percent change of IAP at 24 hours, % | -25.0 ([-33.4]-[-11.4]) | -11.5 ([-23.8]-[0]) |  | 0.018 |
| **Secondary endpoint** |  |  |  |  |
| Increase in stool volume at 24 h after randomization (mL) | 850 (225-1950) | 650 ([-50]-800) |  | 0.01 |
| Increase in stool volume at 7 d after randomization (mL) | 1770 (1225-2330) | 1250 (1000-2300) |  | 0.30 |
| Timing of EN^†^ | 1 (0-3) | 2 (0-3) | — | 0.55 |
| Deterioration of IAH^‡^ | 2 (8.0%) | 5 (21.7%) | 0.37 (0.08-1.72) | 0.18 |
| New-onset ACS | 1 (4.0%) | 3 (13.0%) | 0.31 (0.03-2.74) | 0.27 |
| New-onset organ failure | 7 (28.0%) | 10 (43.5%) | 0.64 (0.29-1.41) | 0.21 |
| Single organ failure |  |  |  |  |
| Respiratory | 1 (4.0%) | 3 (13.0%) | 0.31 (0.03-2.74) | 0.27 |
| Circulatory | 1 (4.0%) | 2 (8.7%) | 0.46 (0.05-4.74) | 0.47 |
| Renal | 0 | 2 (8.7%) | — | 0.22 |
| Multiple organ failure | 5 (20.0%) | 3 (13.0%) | 1.53 (0.41-5.71) | 0.40 |
| Invasive interventions^§^ |  |  |  |  |
| Percutaneous catheter drainage | 4 (16.0%) | 4 (17.4%) | 0.92 (0.26-3.26) | 0.60 |
| Endoscopic transmural drainage | 2 (8.0%) | 2 (8.7%) | 0.92 (0.14-6.01) | 0.66 |
| Endoscopic necrosectomy^¶^ | 0 | 1 (4.3%) | — | 0.48 |
| Surgical laparotomy | 2 (8.0%) | 3 (13.0%) | 0.61 (0.11-3.35) | 0.46 |
| Intra-abdominal bleeding (requiring intervention) | 2 (8.0%) | 3 (13.0%) | 0.61 (0.11-3.35) | 0.46 |
| Enterocutaneous fistula (requiring intervention) | 0 | 0 | — |  |
| Septicemia | 8 (32.0%) | 9 (39.1%) | 0.82 (0.38-1.76) | 0.42 |
| Length of ICU stay (d) | 14 ± 10 | 15 ± 11 |  | 0.77 |
| Death in index hospital stay | 4 (16.0%) | 7 (30.4%) | 0.53 (0.18 -1.56) | 0.31 |
| Length of hospital stay (d) | 23 ± 12 | 22 ± 15 |  | 0.66 |
| Medical expenses (1000 RMB) | 121.3 ± 72.2 | 156.4 ± 146.8 |  | 0.75 |
| Follow up (6 M) | **N = 21** | **N = 16** |  |  |
| Pancreatic pseudocyst | 1 (4.8 %) | 1 (6.3%) | 0.75 (0.04-12.99) | 1.00 |
| Needing elective intervention | 0 | 1 (6.3%) | — | 0.43 |
| Walled-off necrosis | 10 (47.62%) | 6 (37.5%) | 1.52 (0.40-5.71) | 0.74 |
| Needing elective intervention | 2 (10.0%) | 0 | - | 0.50 |
| Portal thrombosis | 1 (4.8 %) | 1 (6.3%) | 0.75 (0.04-12.99) | 1.00 |
| Pancreatogenic portal hypertension | 1 (4.8 %) | 1 (6.3%) | 0.75 (0.04-12.99) | 1.00 |
| New onset diabetes | 8 (29.6%) | 3 (15.8%) | 2.25 (0.51-9.91) | 0.32 |
| Impaired glucose tolerance | 0 | 1 (6.3%) | - | 0.47 |
| External secretion dysfunction | 6 (28.6%) | 2 (12.5%) | 1.69 (0.55-5.17) | 0.42 |
| Recurrent AP | 2 (9.5%) | 1 (6.3%) | 1.87 (0.15-22.94) | 1.00 |
| Death after discharge | 3 (14.3%) | 2 (12.5%) | 1.17 (0.17-7.96) | 1.00 |

ACS, abdominal compartment syndrome; AP, acute pancreatitis; CI, confidence interval; EN, enteral nutrition; IAH, intra-abdominal hypertension; ICU, Intensive Care Unit; RR, relative risk.

^†^Time from randomization to initiation of EN.

^‡^IAP that rebounded ≥ 5 mmHg or increased ≥ 20 mmHg in 1-7 days after randomization.

^§^All interventions after randomization were counted.

^¶^In the conventional group, 1 case underwent percutaneous retroperitoneal endoscopic debridement.

**Table S4. Per-protocol analysis of of primary endpoint and secondary endpoints**

| **Endpoint** | **Neostigmine**  **(n = 40)** | **Conventional**  **(n = 36)** | **RR (95% CI)** | ***P***  **value** |
| --- | --- | --- | --- | --- |
| **Primary endpoint** |  |  |  |  |
| Percent change of IAP at 24 hours, % | -18.7 ([-28.4]-[-4.7]) | -6.8 ([-18.0]-[0]) |  | 0.030 |
| **Secondary endpoint** |  |  |  |  |
| Increase in stool volume at 24 h after randomization (mL) | 870 (250-2080) | 50 ([-50]-780) |  | 0.00 |
| Increase in stool volume at 7 d after randomization (mL) | 1030 (450-1530) | 330 (110-1160) |  | 0.01 |
| Timing of EN^†^ | 2 (0-3) | 2 (0-3) | — | 0.73 |
| Deterioration of IAH^‡^ | 4 (10.0%) | 5 (13.9%) | 0.72 (0.21-2.45) | 0.73 |
| New-onset ACS | 2 (5.0%) | 2 (5.6%) | 0.90 (0.13-6.06) | 1.00 |
| New-onset organ failure | 12 (30.0%) | 14 (38.9%) | 0.77 (0.41-1.44) | 0.47 |
| Single organ failure |  |  |  |  |
| Respiratory | 2 (5.0%) | 6 (16.7%) | 0.30 (0.07-1.39) | 0.14 |
| Cardiovascular | 3 (7.5%) | 1 (2.8%) | 2.70 (0.29-24.81) | 0.62 |
| Renal | 0 (0%) | 3 (8.3%) | — | 0.10 |
| Multiple organ failure | 7 (17.5%) | 4 (11.1%) | 1.68 (0.50-4.94) | 0.52 |
| Invasive interventions^§^ |  |  |  |  |
| Percutaneous catheter drainage | 8 (20.0%) | 5 (13.9%) | 1.44 (0.52-4.00) | 0.55 |
| Endoscopic transmural drainage | 3 (7.5%) | 3 (8.3%) | 0.90 (0.19-4.18) | 1.00 |
| Endoscopic necrosectomy^¶^ | 1 (2.5%) | 2 (5.6%) | 0.45 (0.04-4.76) | 0.60 |
| Surgical laparotomy | 3 (7.5%) | 3 (8.3%) | 0.90 (0.19-4.18) | 1.00 |
| Intra-abdominal bleeding (requiring intervention) | 2 (5.0%) | 4 (11.1%) | 0.45 (0.09-2.31) | 0.41 |
| Enterocutaneous fistula (requiring intervention) | 2 (5.0%) | 0 | — | 0.50 |
| Septicemia | 11 (27.5%) | 9 (25.0%) | 1.10 (0.52-2.35) | 1.00 |
| RAC disease severity |  |  |  |  |
| MSAP | 3 (7.5%) | 5 (13.9%) | 0.54 (0.14-2.10) | 0.47 |
| SAP | 37 (92.5%) | 31 (86.1%) | 1.07 (0.92-1.26) |  |
| Death in index hospital stay | 7 (17.5%) | 5 (13.9%) | 1.26 (0.44-3.26) | 0.75 |
| Length of ICU stay (d) | 14 ± 9 | 15 ± 15 |  | 0.86 |
| Length of hospital stay (d) | 23 ± 13 | 23 ± 17 |  | 0.57 |
| Medical expenses (1000 RMB) | 25.9 ± 83.6 | 129.6 ± 129.9 |  |  |
| **Follow up (6 M)** | **N = 33** | **N = 31** |  |  |
| Pancreatic pseudocyst | 2 (6.1%) | 1 (3.2%) | 1.94 (0.17-22.48) | 1.00 |
| Needing elective intervention | 0 | 1 (3.2%) |  | 0.48 |
| Walled-off necrosis | 14 (42.4%) | 7 (22.6%) | 2.53 (0.85-7.50) | 0.11 |
| Needing elective intervention | 3 (9.1%) | 1 (3.2%) | 3.00 (0.30-30.50) | 0.61 |
| Portal thrombosis | 1 (3.1%) | 1 (3.2%) | 0.94 (0.05-15.67) | 1.00 |
| Pancreatogenic portal hypertension | 1 (3.1%) | 2 (6.5%) | 0.47 (0.04-5.44) | 0.61 |
| New onset diabetes | 9 (27.3%) | 4 (12.9%) | 2.53 (0.69-9.29) | 0.21 |
| Impaired glucose tolerance | 3 (9.1%) | 2 (6.5%) | 1.45 (0.22-9.32) | 1.00 |
| External secretion dysfunction | 7 (24.1%) | 3 (9.7%) | 2.51 (0.59-10.76) | 0.30 |
| Recurrent AP | 4 (12.2%) | 1 (3.2%) | 4.13 (0.44-39.26) | 0.36 |
| Death after discharge | 3 (9.1%) | 3 (9.4 %) | 0.97 (0.18-5.18) | 1.00 |

ACS, abdominal compartment syndrome; AP, acute pancreatitis; CI, confidence interval; EN, enteral nutrition; IAH, intra-abdominal hypertension; ICU, Intensive Care Unit; MSAP, moderately severe acute pancreatitis; RR, relative risk; SAP, severe acute pancreatitis.

^†^Time from randomization to initiation of EN.

^‡^IAP that rebounded ≥ 5 mmHg or increased ≥ 20 mmHg in 1-7 days after randomization.

^§^All interventions after randomization were counted.

^¶^In the neostigmine group, 1 case underwent endoscopic debridement; in the conventional group, 1 case underwent percutaneous retroperitoneal endoscopic debridement and 1 case underwent endoscopic debridement.

**Table S5. Characteristics of patients who developed adverse events**

| **Case** | **Group** | **Sex** | **Age** | **Time from AP onset to ICU (d)** | **Time from AP onset to randomization (d)** | **Etiology of AP** | **Before randomization** | | | **After randomization** | | |
| --- | --- | --- | --- | --- | --- | --- | --- | --- | --- | --- | --- | --- |
|  |  |  |  |  |  |  | **APACHE II** | **Organ failure** | **IAP in mmHg** | **IAP at 9 h in mmHg** | **IAP at 24 h in mmHg** | **IPN** |
| 1 | Neostigmine | M | 38 | 2 | 3 | HTG | 14 | None | 14.70 | 8.80 | 13.24 | Yes |
| 2 | Neostigmine | M | 25 | 3 | 9 | HTG | 8 | Respiratory and Renal | 15.79 | 14.78 | 14.03 | Yes |
| 3 | Neostigmine | M | 57 | 6 | 7 | Alcohol | 12 | Respiratory | 19.85 | 19.12 | 20.59 | Yes |
| 4 | Convention | M | 47 | 2 | 3 | HTG | 14 | Respiratory and Renal | 15.44 | 22.06 | 16.91 | No |
| 5 | Neostigmine | M | 35 | 2 | 3 | HTG | 19 | Respiratory and Renal | 13.97 | 17.65 | 18.38 | Yes |
| 6 | Neostigmine | M | 61 | 1 | 3 | Biliary | 14 | None | 22.05 | 13.24 | 22.60 | No |

AP, acute pancreatitis; APACHE II, Acute Physiology and Chronic Health Evaluation II; IAP, intra-abdominal pressure; ICU, Intensive Care Unit; IPN, infected pancreatic necrosis; HTG, hypertriglyceridemia.

**Table S6. Adverse events, causes and outcomes**

| **Cases** | **Adverse event** | **Occurrence after randomization (d)** | **Action taken** | **Relationship to neostigmine** | **Possible causes of adverse events**  **determined by clinicians** | **Hospital**  **Stay (d)** | **ICU stay (d)** | **Outcome** |
| --- | --- | --- | --- | --- | --- | --- | --- | --- |
| 1 | Bradycardia | 4 | Withdrawal of esmolol | Unlikely | The bradycardia may be caused by esmolol, a beta-adrenergic receptor blocker. After the withdrawal of esmolol, the heart rate rose to 90 beats/min, while neostigmine was not stopped. | 19 | 12 | Survived |
| 2 | Circulatory failure | 4 (5 h) | Fluid resuscitation, norepinephrine | Unlikely | The patient received mechanical ventilation and was sedated with midazolam and dexmedetomidine which may cause the decrease of blood pressure. | 25 | 25 | Deceased |
| 3 | Circulatory failure | 6 | Fluid resuscitation, norepinephrine, antibiotics, PCD and endoscopic debridement | Unlikely | The new onset of circulatory failure may be caused by IPN. The patient received PCD in the second week and *Klebsiella pneumoniae* was cultured in the blood during the third week. | 36 | 36 | Survived |
| 4 | Circulatory failure | 1 (24 h) | Fluid resuscitation, norepinephrine, | Unlikely | Circulatory failure may be induced by persistent SIRS. | 5 | 5 | Deceased |
| 5 | Circulatory failure | 13 | Fluid resuscitation, norepinephrine, antibiotics and PCD | Unlikely | The new onset of circulatory failure was most likely due to infection. The patient had persistent high fever, IPN and received PCD intervention and died of septic shock one month after disease onset. | 28 | 28 | Deceased |
| 6 | Respiratory failure | 15 | Mechanical ventilation | Unlikely | The new onset of respiratory failure was due to ACS. The patient developed dyspnea during IAP rebound and died of multiple organ failure 72 h thereafter. | 4 | 4 | Deceased |

ACS, abdominal compartment syndrome; IAP, intra-abdominal pressure; ICU, Intensive Care Unit; IPN, infected pancreatic necrosis; PCD, percutaneous catheter drainage; SIRS, Systemic Inflammatory Response Syndrome.
